# The role of gender as a barrier to the professional development of psychiatrists

**DOI:** 10.1192/j.eurpsy.2023.2462

**Published:** 2023-10-18

**Authors:** Ozge Kilic, Anita Riecher-Rössler, Silvana Galderisi, Philip Gorwood, Sophia Frangou, Mariana Pinto da Costa

**Affiliations:** 1 Department of Psychiatry, Bezmialem Vakif University Faculty of Medicine, Istanbul, Turkey; 2 Medical Faculty, University of Basel, Basel, Switzerland; 3 Department of Mental and Physical Health and Preventive Medicine, University of Campania Luigi Vanvitelli, Napoli, Italy; 4 Université Paris Cité, GHU Paris Psychiatrie et Neurosciences, CMME, Hôpital Sainte-Anne, Paris, France; 5 Institute of Psychiatry and Neuroscience of Paris (IPNP), INSERM U1266, Paris, France; 6 Department of Psychiatry, Icahn School of Medicine at Mount Sinai, New York, NY, USA; 7 Djavad Mowafaghian Centre for Brain Health, University of British Columbia, Vancouver, BC, Canada; 8 South London & Maudsley NHS Foundation Trust, London, UK; 9 Institute of Psychiatry, Psychology & Neuroscience, King’s College of London, London, UK; 10 Institute of Biomedical Sciences Abel Salazar, University of Porto, Porto, Portugal

**Keywords:** bias, discrimination, gender, sex, sexual harassment, women

## Abstract

**Background:**

Despite efforts toward greater gender equality in clinical and academic psychiatry in recent years, more information is needed about the challenges in professional development within psychiatry, and how these may vary with gender.

**Methods:**

A cross-sectional 27-item online survey was conducted with psychiatrists and psychiatric trainee members of the European Psychiatric Association.

**Results:**

A total of 561 psychiatrists and psychiatric trainees from 35 European countries participated representing a response rate of 52.8% for women and 17.7% for men from a total sample of 1,580. The specific challenges that women face in their professional development fall into two categories. One comprised women’s negative attitudes concerning their abilities in self-promotion and networking. The other identified environmental barriers related to lack of opportunity and support and gender discrimination. Compared to men, women reported higher rates of gender discrimination in terms of professional advancement. Women were less likely to agree that their institutions had regular activities promoting inclusion, diversity, and training to address implicit gender bias. Working in high-income countries compared to middle-income countries relates to reporting institutional support for career progression.

**Conclusions:**

These findings are an open call to hospital leaders, deans of medical schools, and department chairs to increase efforts to eradicate bias against women and create safer, inclusive, and respectful environments for all psychiatrists, a special call to women psychiatrists to be aware of inner tendencies to avoid self-promotion and networking and to think positively and confidently about themselves and their abilities.

## Introduction

In recent decades, psychiatry has had one of the highest percentages of women enrolling in training programs among medical specialties. However, the ratio of women to men in leadership roles is still disproportionally low [1, 2]. According to the She Figures data 2021 [3], the proportion of women academic staff in Science, Technology, Engineering, and Mathematics in the European Union declined with increasing seniority from 34.9% in grade C positions (the first grade into which a newly qualified Ph.D. graduate would normally be recruited) to 28.2% in grade B positions (positions between C and A) and less than 20% of staff in grade A positions (single highest grade at which research is conducted within the institutional system). The discrepancies in women’s representation among grade A staff at the national level substantially mirror the patterns observed at the European level [4].

Women do worse than comparably qualified men in salary, promotions, grants, and scholarly publishing [5]. In 2017, an article in European Psychiatry highlighted the gender gap in publishing in three highest-impact psychiatric journals [6]. A further report in 2019, which encompassed all psychiatric literature between 2008 and 2018 (30,934 articles), found near gender parity for the first authors but lower rates of women (% 20–25) in senior authors positions [7].

In terms of clinical and managerial leadership positions, a study by the UK National Health Service (NHS) found that more than half of senior managers in the NHS are men, despite more than 75% of the workforce being women [8].

Several external factors impeding women physicians’ advancement to leadership positions have been described: (i) lack of equal opportunities in recruitment, hiring, and promotion [9], (ii) lack of mentorship and sponsorship programs [10, 11], (iii) lack of leadership development, (iv) institutional environment, gender-based discrimination, and harassment [12], (v) fewer opportunities for networking and collaboration [13], and (vi) more work-life responsibilities [14]. Other barriers reflect (i) sex-role orientation [15], (ii) negative self-views of the effectiveness of women leaders [16], and (iii) prejudicial evaluations of women’s competence as leaders [17, 18]. The glass ceiling effect can be the overall result of these barriers. In particular, barriers to the professional development of women in psychiatry have been less studied.

This study represents the continuous efforts of the European Psychiatric Association (EPA) to address barriers and challenges that are more common for women than men in professional life, to increase awareness within Europe, and to identify national variations.

## Methods

### Study design, settings, and participants

We conducted a cross-sectional survey of psychiatrists and psychiatry trainees working in any European country who were registered with the EPA as members. Potential participants were emailed a link to the survey through SurveyMonkey.

### Instrument

We used a 27-item questionnaire developed by the study team, which included items adapted and modified from previous study instruments used by the Society of Biological Psychiatry and the International Society for Bipolar Disorders (ISBD). Items measured self-confidence in one’s abilities, willingness to engage in and attitudes toward self-promotion (8 items); perceived level of career support, gender discrimination, and protection from harassment at their current institution (10 items), and attitudes and engagement with networking (9 items). Items were presented in the form of statements, and participants were asked to indicate their agreement on a 9-point scale from 0 (completely disagree) to 8 (completely agree). Respondents were also asked to provide information about their age, gender, professional degree(s), the current field of work defined as adult psychiatry (AP) or child and adolescent psychiatry (CAP) or other, the country they currently work, and years of professional experience in the current field (including post-graduate training).

### Statistical analysis

Data were analyzed using SPSS 26.0 [19]. Demographic and professional characteristics of participants were described using simple frequency distributions and compared between genders using the Mann–Whitney U test or the Chi-Square test according to the type of data. Three socioeconomic data points were missing (1 from age; 2 from professional degree) while gender, current field, years of professional experience, and country of work did not have any missing values. In each of the three blocks of items (self-promotion, current institutional environment, and networking), the missing data percentage was the same at 31%.

The Likert-type scores from 0 (totally disagree) to 8 (totally agree) of survey items were grouped into three categories. Scores between 0 and 3 were categorized as “disagree,” scores 4 and 5 were categorized as “neither agree nor disagree,” and scores 6 and 8 were categorized as “agree” and compared between women and men with the Chi-Square test. We opted not to correct for multiple comparisons as this survey was not designed for hypothesis testing but to explore gender differences in external and internal barriers to career advancement.

Items that significantly differed between men and women were entered as the dependent variable in separate linear regression analyses conducted to examine the relationship with gender. For the linear model, gender was coded as the dummy variable with men as the reference category (coded as 0) and women as the comparison category (coded as 1), the professional degree was recoded as a professor (1) and not a professor (0), and subspecialty was recoded as AP (0) and CAP (1). Countries of current work were grouped according to middle-income and high-income countries [20]. Age, gender, years of professional experience, professional degree, current field, and country according to income were entered simultaneously into the linear regression model. The significance level was set as p<0.05.

### Ethical considerations

This study was conducted per the principles of good scientific practice. Eligible respondents were given written information about the aim of the study when invited to participate. Consent was implied if the questionnaire was completed. Privacy was maintained through the SurveyMonkey option of not logging the IP address making it impossible to link individual surveys back to the identity of respondents.

## Results

### Responder characteristics

A total of 561 participants from 35 countries in Europe took part in this study (age range 24–88 years). The total target sample was 1,580 with a total response rate of 35.5% (representing a response rate of 52.8% for women and 17.7% for men). The distribution of participants by country is shown in Table 1. Data from participants who self-identified as non-binary were excluded from the statistical analysis of group comparisons due to their small number (N=3). The mean age (standard deviation) of the sample was 36.05 (9.60) years.

**Table 1.** The distribution and percentage of participants by country

	Country where you work	Percentages of responses	Total no. of responses
1	Austria	1.25	7
2	Azerbaijan	2.85	16
3	Belarus	1.60	9
4	Belgium	1.25	7
5	Bosnia and Herzegovina	1.25	7
6	Bulgaria	0.36	2
7	Croatia	1.60	9
8	Czech Republic	0.71	4
9	Denmark	1.96	11
10	Estonia	0.36	2
11	Finland	1.60	9
12	France	4.46	25
13	Georgia	1.43	8
14	Germany	2.14	12
15	Greece	3.21	18
16	Hungary	0.89	5
17	Ireland	0.53	3
18	Israel	0.18	1
19	Italy	5.53	31
20	Latvia	0.18	1
21	Malta	0.71	4
22	Netherlands	0.71	4
23	Norway	1.25	7
24	Poland	5.35	30
25	Portugal	6.77	38
26	Romania	8.56	48
27	Russian Federation	7.13	40
28	Serbia	1.25	7
29	Slovakia	0.18	1
30	Spain	0.89	5
31	Sweden	1.60	9
32	Switzerland	3.74	21
33	Turkey	17.11	96
34	Ukraine	2.85	16
35	United Kingdom of Great Britain and Northern Ireland	7.66	43
36	Other European countries	0.89	5

**Figure 1.**
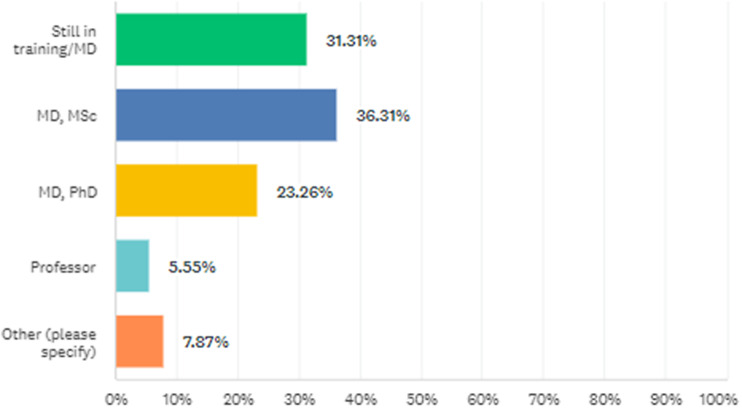
The percentage of participants according to professional degrees.

The percentage of participants according to professional degrees is shown in Figure 1. Regarding the current field of work, 505 participants practiced AP, 55 practiced CAP, and 1 had dual accreditation. Years of professional experience in the current field were 8.5 (8.2) and 6.00 (1–62) as mean (standard deviation) and median (range).

**Figure 2.**
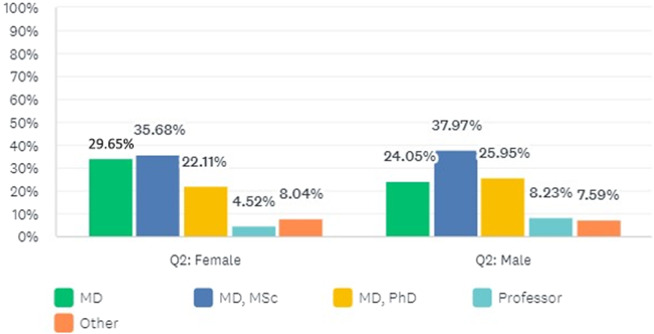
The distribution of men and women according to professional degrees.

There were no significant differences among genders in terms of the distribution of professional degrees (*p* = 0.436; Figure 2). There were significant differences between genders in terms of the distribution of the current fields of work defined as AP, CAP, and others (*p* = 0.001). The proportion of women in CAP was higher than the proportion of men in CAP. Characteristics of participants by gender are shown in Table 2.

**Table 2.** Characteristics by gender

	Women (*n* = 399)	Men (*n* = 159)	*p*
Age (median) (range)	34 (24–78)	34 (25–88)	0.507
Years of professional experience (median) (range)	6 (1–45)	6 (1–62)	0.301
Current field (%)			
Adult psychiatry	82.5	91.2	0.001
Child and adolescent psychiatry	12.5	2.5	
Other	5.0	6.3	

### Gender differences in the survey results

A higher proportion of women (31%) compared to men (15%) reported that they do not have the skills necessary to effectively promote their accomplishments (*p* = 0.005) (Table 3).

**Table 3.** Responses to items on self-promotion by gender

	Men (%)	Women (%)	*χ* ^2^	*df*	*p*
I feel that I do not have to self-promote, as my work will speak for itself			0.595	2	0.743
Disagree	43	44.2			
Neither agree or disagree	28.5	24.8			
Agree	28.5	31			
My job is to help patients, teach, advocate, or do research, not show off my own accomplishments			0.680	2	0.712
Disagree	17.7	15.7			
Neither agree or disagree	19.5	23			
Agree	62.8	61.3			
People who talk about their own successes and accomplishments are just trying to get praise and feed their egos			2.521	2	0.284
Disagree	37.2	35			
Neither agree or disagree	29.2	37.2			
Agree	33.6	27.7			
It is not my place to promote the work I do since more senior members of the team should get the credit			1.689	2	0.430
Disagree	60.2	66.4			
Neither agree or disagree	27.4	21.6			
Agree	12.4	12			
In my culture, it is not acceptable to brag about one’s accomplishments			4.226	2	0.121
Disagree	33.6	32.8			
Neither agree or disagree	44.2	35.4			
Agree	22.1	31.8			
I do not feel comfortable promoting myself because of how it will make me look to others			5.439	2	0.66
Disagree	31	21.5			
Neither agree or disagree	32.7	30.7			
Agree	36.3	47.8			
When I have promoted myself, people have reacted negatively, making me not want to try again			4.210	2	0.122
Disagree	56.6	45.2			
Neither agree or disagree	28.4	36.9			
Agree	15	17.9			
I do not have the skills necessary to effectively promote my accomplishments			10.544	2	**0.005**
Disagree	46.9	38.7			
Neither agree or disagree	38.1	30.3			
Agree	15	31			

A higher proportion of women (27%) compared to men (9.7%) reported that they had experienced discrimination in terms of professional advancement because of their gender (*p* < 0.001). A lower proportion of women (4%) compared to men (13.3%) agreed that their institution provided training to address implicit bias (*p* = 0.002). A lower proportion of women (14.6%) compared to men (34.5%) agreed that their institution provided training to deal with sexual harassment (*p <* 0.001). A lower proportion of women (6.5%) compared to men (18.6%) reported that their institution has regular activities to promote diversity and inclusion relevant to women (*p <* 0.001) (Table 4).

**Table 4.** Responses to items on institutional environment by gender

	Men (%)	Women (%)	*χ* ^2^	*df*	*p*
I have access through my institution to training activities to improve my presentation and self-promotion skills			2.658	2	0.265
Disagree	51.3	58			
Neither agree or disagree	29.2	21.5			
Agree	19.5	20.5			
I have access through my institution to training activities to improve my networking skills			3.800	2	0.150
Disagree	54.9	61.7			
Neither agree or disagree	29.2	20.1			
Agree	15.9	18.2			
I have access through my institution to a personal mentor			0.240	2	0.887
Disagree	48.7	49.7			
Neither agree or disagree	21.2	22.6			
Agree	30.1	27.7			
I am satisfied with the support I receive from my mentor			0.377	2	0.828
Disagree	39.8	43.1			
Neither agree or disagree	30.1	29.2			
Agree	30.1	27.7			
My institution provides support (e.g., time off, financial aid) to attend activities relevant to my career progression			4.632	2	0.099
Disagree	38.1	49.3			
Neither agree or disagree	29.2	26.6			
Agree	32.7	24.1			
My institution has regular activities to promote diversity and inclusion relevant to women			21.355	2	**<0.001**
Disagree	54	76.3			
Neither agree or disagree	27.4	17.2			
Agree	18.6	6.5			
My institution has clear policies to deal with sexual harassment			23.014	2	**<0.001**
Disagree	38.1	60.2			
Neither agree or disagree	27.4	25.2			
Agree	34.5	14.6			
My institution provides training to address implicit bias			12.353	2	**0.002**
Disagree	61.9	74.5			
Neither agree or disagree	24.8	21.5			
Agree	13.3	4			
I have experienced discrimination in terms of professional advancement because I am a woman/man			29.077	2	**<0.001**
Disagree	61.9	74.5			
Neither agree or disagree	24.8	21.5			
Agree	13.3	4			
I have experienced sexual harassment at my institution			4.612	2	0.100
Disagree	85	77.7			
Neither agree or disagree	10.6	10.9			
Agree	4.4	11.3			

The significance level was set as p<0.05.

A higher proportion of women (21.9%) compared to men (13.3%) reported that they do not have the skills necessary to effectively create and maintain professional networks (*p* = 0.028) (Table 5).

**Table 5.** Responses to items on networking by gender

	Men (%)	Women (%)	*χ* ^2^	*df*	*p*
It is what you do, not who you know, that leads to success					
Disagree	38.9	48.2	2.762	2	0.251
Neither agree or disagree	30.1	25.2			
Agree	31	26.6			
It is my mentor/team leader’s responsibility to introduce me to the people I need to meet professionally					
Disagree	53.1	53.6	1.178	2	0.555
Neither agree or disagree	27.4	31			
Agree	19.5	15.3			
I am too junior or inexperienced to approach more senior people in my field or my institution					
Disagree	69	61.3	2.057	2	0.357
Neither agree or disagree	16.8	20.8			
Agree	14.2	17.9			
I am shy and introverted, so meeting other professionals is not worth the effort or anxiety					
Disagree	62.8	56.6	3.771	2	0.152
Neither agree or disagree	25.7	23.7			
Agree	11.5	19.7			
When I approach people for networking purposes, I feel as if I am using them for my own advancement and it is awkward because they can tell that my motives are not purely social					
Disagree	53.1	46	3.975	2	0.137
Neither agree or disagree	32.7	31			
Agree	14.2	23			
As a wo(man), I feel it is potentially unsafe to approach strangers (particularly more senior wo(men) in order to establish new professional connections for risk of the overture being misunderstood)					
Disagree	73.5	60.6	9.371	2	**0.009**
Neither agree or disagree	21.2	23.4			
Agree	5.3	16.1			
The networks that could be of most use to me are not willing to let me in (e.g., old boys’ club, old women’s club)					
Disagree	56.6	52.2	1.718	2	0.424
Neither agree or disagree	35.4	35.4			
Agree	8	12.4			
I do not have the skills necessary to effectively create and maintain professional networks					
Disagree	65.5	51.1	7.118	2	**0.028**
Neither agree or disagree	21.2	27			
Agree	13.3	21.9			
I do not have the skills necessary to effectively promote my accomplishments					
Disagree	61.9	42.3	13.64	2	**0.001**
Neither agree or disagree	23.9	29.9			
Agree	14.2	27.7			

The significance level was set as p<0.05.

### Factors influencing the reporting of low self-promotion skills

Gender, age, years of professional experience, current field, professional degree, and the country of current work as independent variables, and not having the skills to effectively promote accomplishments as the dependent variable were entered into a linear regression model, which was statistically significant (*R* = 0.20, *F* = 2.572, *R^2^* = 0.04, *df* = 6; *p* = 0.019). Results revealed that only gender had a significant effect on reports of lacking skills (standardized coefficients – beta) (*b* = 0.108) (*p* = 0.035).

### Factors influencing reports of gender discrimination for professional advancement

The overall linear regression model with all demographic and professional characteristics as independent and reports of experience of discrimination due to gender as the dependent variable was statistically significant (*R* = 0.33, *F* = 7.351, *R^2^* = 0.11, *df* = 6; *p <* 0.001). Results revealed a significant effect of being women on reports of experiencing discrimination (*b* = 0.31) (*p <* 0.001) but no significant effects of age, years of professional experience, current field, professional degree, or country of current work.

### Factors influencing reports about the level of institutional support for career progression

The linear regression model with reports on institutions providing support for activities relevant to career progression as the dependent variable was statistically significant (*R* = 0.24, *F* = 3.759, *R^2^* = 0.06, *df* = 6; *p* = 0.001). Working in a high-income compared to a middle-income country had a significant effect on reporting institutional support (e.g., time off, financial aid) for career progression (*b* = 0.19) (*p <* 0.001). No other factors significantly affect this outcome.

### Factors influencing reports of regular institutional activities promoting inclusion and diversity relevant to women

The linear regression model with all demographic and professional characteristics as independent and reports on institutions promoting inclusion and diversity as the dependent variable was statistically significant (*R* = 0.33, *F* = 7.365, *R^2^* = 0.11, *df* = 6; *p* < 0.001). Older age (*b* = 0.27) (*p* = 0.014) and being men compared to women (*b* = − 0.24) (*p <* 0.001) were related to reports that institutions had regular activities to promote diversity and inclusion relevant to women. Current field, years of professional experience, professional degree, and country of work did not affect this outcome.

**Figure 3.**
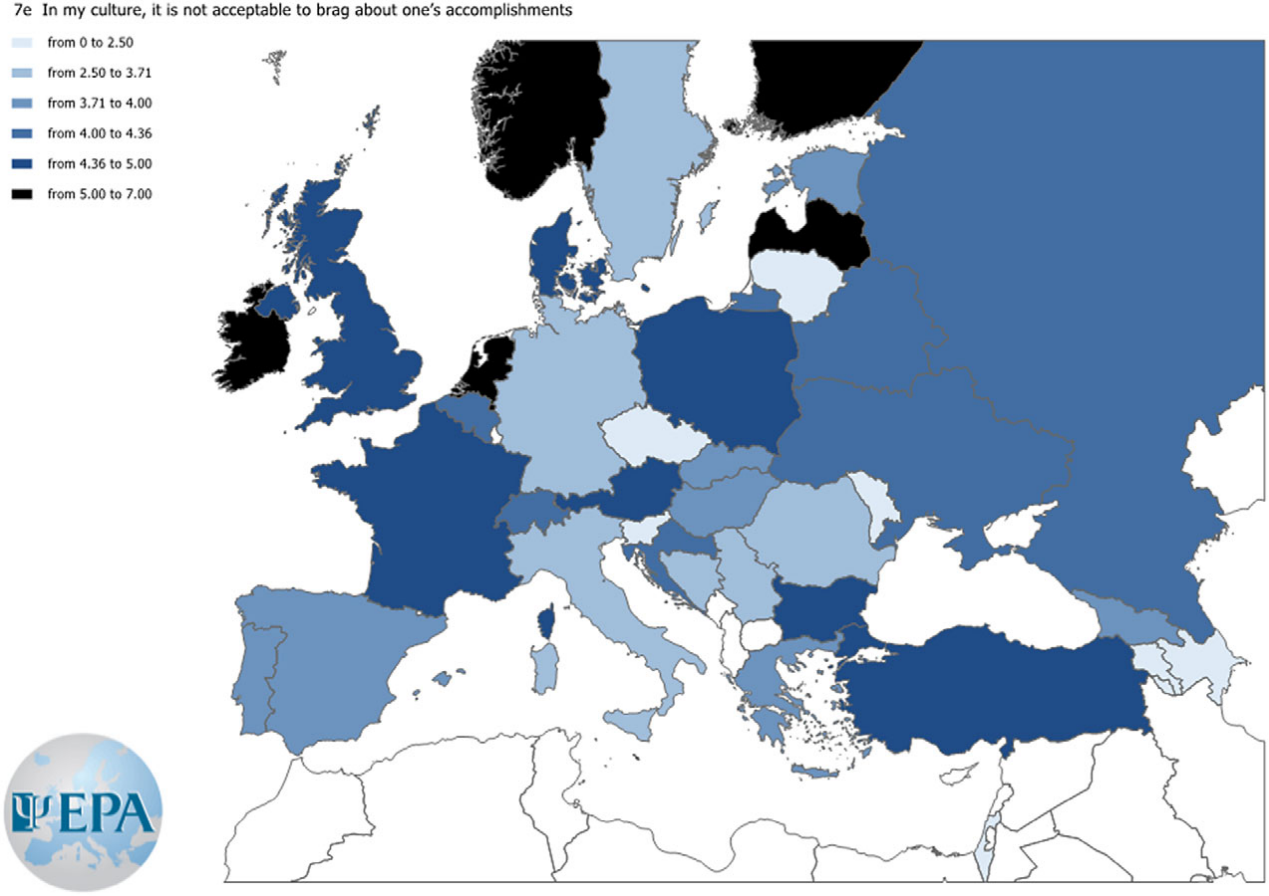
Agreement level to the item “In my culture, it is not acceptable to brag about one’s accomplishments.”

**Figure 4.**
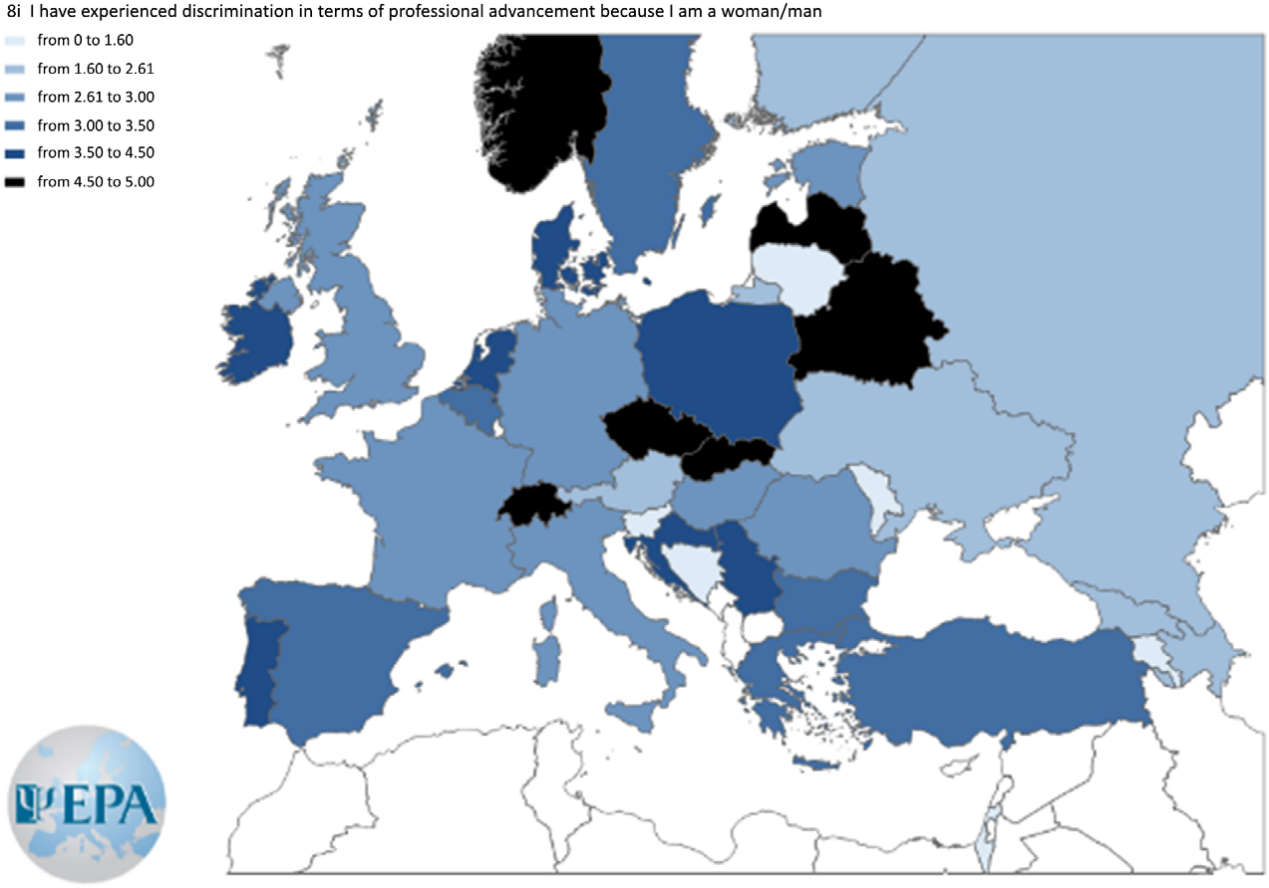
Agreement level to the item “I have experienced discrimination in terms of professional advancement because I am a woman/man.”

**Figure 5.**
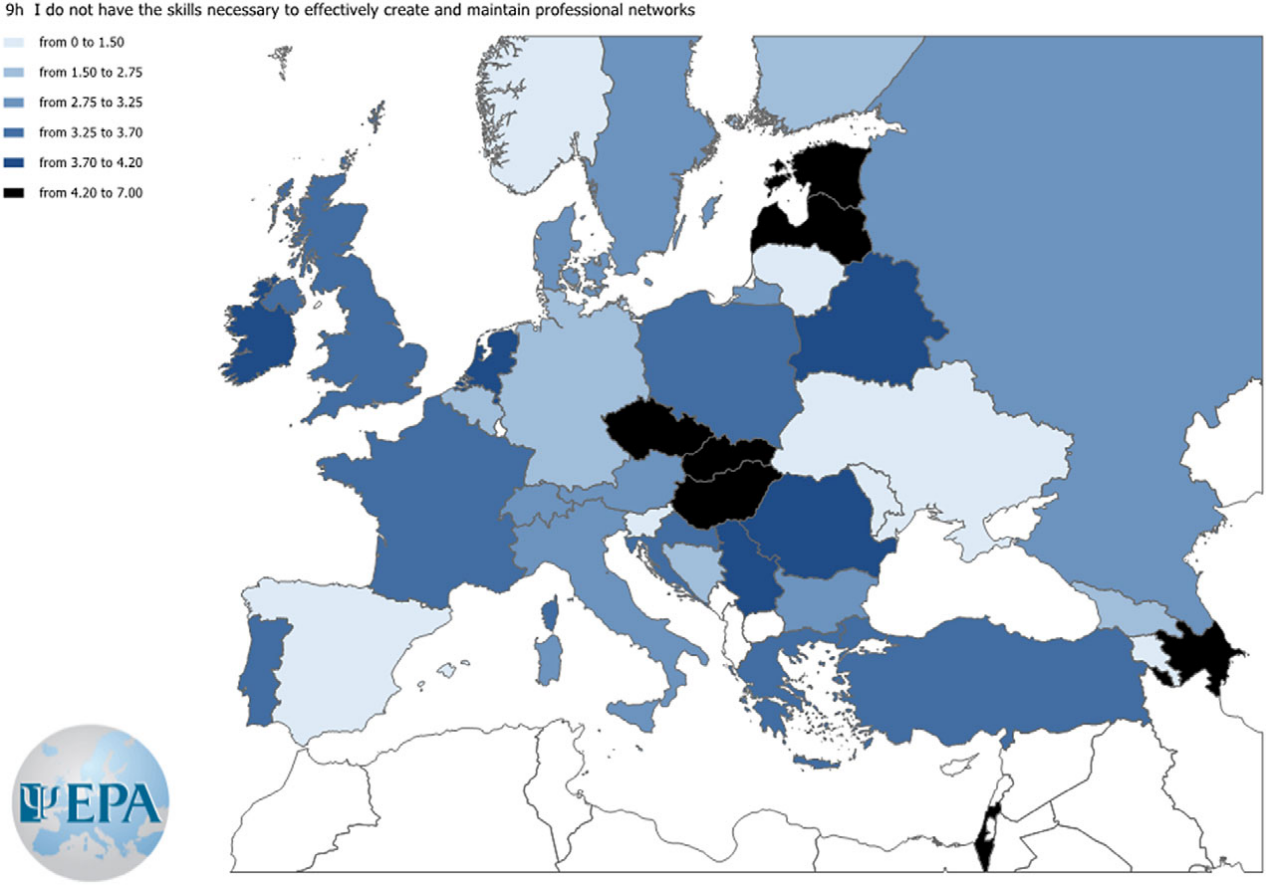
Agreement level to the item “I do not have the skills necessary to effectively create and maintain professional networks.”

An item that samples each of the item blocks “self-promotion,” “institutional environment,” and “networking” was chosen. Three maps were built to show responses to these items across countries according to the Likert-type scale (0–8). Agreement levels for the three items selected from each block are displayed in Figures 3–5.

### Factors influencing reports of clear institutional policies for sexual harassment

The linear regression was statistically significant (*R* = 0.35, *F* = 8.832, *R^2^* = 0.12, *df* = 6; *p* < 0.001). Men compared to women (*b* = − 0.23) (*p <* 0.001), working in high-income compared to middle-income countries (*b* = 0.17) (*p* = 0.001), and increasing age (*b* = 0.32) (*p* = 0.003) were related to this outcome. Current field, years of professional experience, and professional degree did not affect this outcome.

### Factors influencing reports of institutional provision of implicit bias training

The linear regression was statistically significant (*R* = 0.29, *F* = 5.716, *R^2^* = 0.08, *df* = 6; *p* < 0.001). Men compared to women (*b* = − 0.17) (*p* < 0.001), working in high-income compared to middle-income countries (*b* = 0.12) (*p* = 0.016), and increasing age (*b* = 0.31) (*p* = 0.004) were significantly related. Current field, years of professional experience, and professional degree did not affect this outcome.

### Factors influencing reports of lower networking skills

The linear regression was statistically significant (*R* = 0.27, *F* = 4.763, *R^2^* = 0.07, *df* = 6; *p* < 0.001). Women compared to men (*b* = 0.32) (*p* = 0.032) were significantly related. Age, current field, years of professional experience, and professional degree did not affect this outcome.

## Discussion

This international study with a sample of 561 psychiatry trainees and psychiatrists from 35 European countries identified specific challenges that women face in their professional development that fell into two categories. One comprised women’s negative attitudes regarding their abilities in self-promotion and networking. The other identified environmental barriers relate to lack of opportunity and support and gender discrimination. Although our results do not show a direct relationship, negative beliefs about self-promotion and networking, and the quality of the institutional environment and opportunities could be related to the inequality in leadership positions.

### Barriers associated with women’s attitudes

Women psychiatrists compared to men were more likely to report not having the necessary skills to promote their accomplishments and to create and maintain professional networks. Similarly, prior research showed that women physicians involved in clinical research consistently evaluated themselves as being less capable than men in performing or applying their knowledge and skills [21]. Several explanations were proposed to explain these findings. First, the backlash avoidance theory postulates that women fear the negative social consequences of self-promotion [22]. Another explanation relates to the “confidence gap” phenomenon. A study that examined the written narrative evaluations of internal medicine trainees found that, compared to men, women trainees were more likely to be described as lacking confidence by their faculty even after adjusting for faculty gender, numeric rating, and post-graduate year [23]. A further explanation relates to the “modesty norm,” which refers to expectations, across cultures, that women should “be nice” and “not too demanding” [24]. The impostor syndrome is a pattern of self-doubt coupled with fears of being exposed as a fraudster despite evidence of achievements. It was shown to affect half of females compared to one-fourth of male students [25]. These tendencies and their consequences may be addressed by both raising awareness among women and providing training opportunities that improve self-confidence and self-promotion skills.

### Barriers associated with the institutional environment

Working in a high-income country compared to a middle-income country was related to reporting greater institutional support activities relevant to career progression (e.g., time off, financial aid) possibly reflecting the level of resources available.

Women reported having experienced higher levels of gender discrimination than men in terms of professional advancement in their institutional environment. The gender gap in experiences of the majority of forms of harassment narrows as women’s representation in a field of study rises [26]. Findings from a systematic review showed that 27% of women surgical trainees had experienced sexual harassment – much higher than men with 5%. Of those, 71% did not notify their institution about these behaviors. Fifty-one percent said it was because they were afraid of retaliation. Over half (56%) of those who reported their experiences said they had a negative experience reporting [27].

Women were more likely to view institutional activities that address implicit bias, deal with sexual harassment, and promote diversity and inclusion relevant to women as insufficient. Increasing age raised the likelihood of reporting that institutions (i) provided training to address implicit bias, (ii) had clear policies to deal with sexual harassment, and (iii) had regular activities to promote diversity and inclusion relevant to women. One explanation could be that older psychiatrists compared and judged the current conditions to their earlier experiences in the field, which were even worse.

Previous research identified institutional impediments to mentoring, time management, the influence of bias, exclusion from official and informal networks, and lack of participation in committees and non-promotion activities as barriers to women’s advancement in academic medicine and academic psychiatry [28]. Moreover, women faculty, especially those in clinical positions, have expressed concerns about role overload, citing factors like the demands of their current positions or the addition of new roles as obstacles to advancement, no matter how desirable those new roles may be. Women’s barriers were mostly internal (“I can’t do it anymore”), while men’s barriers were mostly external (“leaders are impeding my progress”) [29].

### Strengths and limitations

The main limitation of the study is convenience sampling and the potential for socio-desirability bias in responding. The sample size being relatively small considering the number of psychiatric trainees and psychiatrists in Europe may limit broad generalizations. Beliefs, perceptions, and behaviors are complex constructs, and both men and women show individual differences influenced by the personal and professional experiences within each gender group.

### Implications of the findings for future policies, practice, and research

#### Changing women’s attitudes

Self-promotion and networking skills are useful throughout a career path to gain recognition and advance professionally. Raising awareness on gender-related issues through research, publications, and sharing of information at congresses, web pages, and social media platforms [30] could be a first step. International and national psychiatric associations may provide advice and act as a hub for initiatives and facilitate collaborations.

Training activities during undergraduate and postgraduate psychiatric education on these skills are one of the essential solutions to dismantle barriers to professional development. Gender equity in training opportunities may be maintained through sustainable, strategic programs that encourage networking and mentoring, both informally and through formal collaborations [5] and career development initiatives [6]. It is crucial to create policies and processes for professional advancement such as providing mentorship and assistance for women as well as to recognize the time spent in mentorship [31].

Post-graduate education is another platform that should promote gender equity. Educational programs should include learning activities that address gender differences when preparing physicians for careers [21].

Institutional leadership must commit to eradicating harassment and bias. In academic medicine, harassment identification and response training as well as a systemic transformation of cultures that target harassment and reprimand these behaviors are all required to create safer environments for all academic physicians [32]. Training and awareness about implicit and explicit bias expressions and interventions, the creation of diverse, inclusive, and respectful environments, and integration of these entities into policies and procedures may be especially beneficial [32].

Establishing equity, diversity, and inclusion committees to track and hold institutions accountable for systemic discriminatory practices, cultivating supportive cultures where colleagues feel empowered to speak out against and report harassment, and implementing open door and zero tolerance policies that affirm experiences of women and target retaliation or other negative outcomes may all help to intentionally create safer and more equitable workplaces [32, 33]. In its 2021 policy action paper on closing the leadership gap in health care, the World Health Organization outlined four strategic aspects in its framework to promote women leadership: lay the groundwork for equality, identify social norms and preconceptions, address workplace systems and cultures, and empower women to achieve [34]. The purpose of diversity, equity, and inclusion committees, bureaus, or individuals assigned to handle these issues is to institutionally address harassment and discrimination and bring these issues to the foreground regularly, empowering the community to lessen, prevent, and report sexual harassment [35]. Training that specifically targets harassment and implicit bias is a crucial component. Most academic institutions offer digital anti-bias and anti-harassment training modules. Often these are “check the box” efforts to lessen harassment and bias, but they fall short of the critical group dialogue required to strengthen learning and encourage the self-reflection required to transform thinking and behavior. Mandatory training for all levels of leadership and faculty, as well as regular review, is required. Furthermore, assessing outcomes, including external feedback, is critical for determining whether harassment and bias incidents have decreased, as well as for analyzing whether institutional and departmental anti-bias and anti-harassment goals have been met [35].

Promoting women to leadership positions could be through: (i) exposing to women who can provide positive role models [36]; (ii) the availability of practical advice regarding positive attitudes and behaviors for building successful career strategies, and (iii) working together with men to identify attitudinal and institutional barriers. Gender parity is not a “women’s issue” only. A key report, entitled “Men as Allies” published in 2019 in the UK, highlighted the fact that progress in closing the gender gap can only be accelerated if men are also actively involved in addressing barriers to leadership [37].

There are several noteworthy initiatives to support the career development of women psychiatrists. The EPA has created the Constance Pascal–Helen Boyle Prize for Outstanding Achievement by a Woman in Working to Improve Mental Health Care in Europe [38]. The American College of Neuropsychopharmacology has a dedicated women’s task force that surveys women’s needs and organizes specific events during its annual meetings. The women’s luncheon is a landmark event in these meetings [39]. The Society for Biological Psychiatry, through its Women’s Leadership Group [40] and The Women’s Initiative of the ISBD [41], has adopted a similar model. The Women and Mental Health Special Interest Group of the Royal College of Psychiatrists plays a dual role in advocating for mental health services for women and in promoting careers of women psychiatrists toward academic, clinical, and managerial leadership positions [42].

Although this study did not enquire about the marital and parenting status of participants, another barrier to the professional advancement of women is their greater involvement in parenting, caring, and homemaking activities [43]. Women still have a higher probability than their male counterparts to reduce their careers ambitions for their children starting most commonly with maternal leave [18, 44] and reduced working hours [45]. Self-reported discrimination based on pregnancy, maternity leave, or breastfeeding was deemed to be maternal discrimination and was reported in more than one-third of physician mothers [46]. Future research should further investigate this.

Our findings are an open call to hospital leaders, deans of medical schools, and department chairs to support a change to eradicate harassment and bias and create safer, diverse, inclusive, and respectful environments for all genders. And a call to the partners of young psychiatrists to share the burden of homemaking and parenting equally. And, last not least, a special call to women psychiatrists to notice inner tendencies to avoid self-promotion and networking and start positively regarding themselves and their skills, and to critically reflect own traditional gender role behavior.
